# Exosomes from adipose‐derived stem cells regulate macrophage polarization and accelerate diabetic wound healing via the circ‐Rps5/miR‐124‐3p axis

**DOI:** 10.1002/iid3.1274

**Published:** 2024-06-18

**Authors:** Dongjing Yin, Guoliang Shen

**Affiliations:** ^1^ Department of Burns and Plastic Surgery The First Affiliated Hospital of Soochow University Suzhou Jiangsu China; ^2^ Department of Burns and Plastic Surgery Affiliated Nantong Hospital 3 of Nantong University Nantong Jiangsu China

**Keywords:** ADSCs, diabetes, exosomes, macrophage polarization, wound healing

## Abstract

**Background:**

Adipose‐derived stem cells (ADSCs) hold promising application prospects in the treatment of diabetic wounds, although the underlying mechanisms of repair have not been fully elucidated. This research aimed to elucidate the mechanisms by which ADSCs promote wound healing.

**Methods:**

Exosomes from ADSCs were isolated and circRps5 level was identified. To investigate the role of circRps5 in the regulation, exosomes from differently treated ADSCs were used. Different exosomes were injected into the edge of the wound in diabetic mice, and the effects on wound healing status, pathology, collagen, cytokines, and macrophage phenotype were assessed. Raw264.7 cells were co‐treated with high glucose and exosomes, and then cell phenotype and autophagy were examined in vitro, followed by the evaluation of miR‐124‐3p's impact on cell phenotype.

**Results:**

Exosomes from ADSCs were isolated and identified using nanoparticle tracking analysis and exosome markers. Overexpression of circRps5 accelerated wound healing, reduced inflammatory response, enhanced collagen production, and promoted the M2 transformation of macrophages. In high glucose‐induced macrophages, its overexpression also inhibited excessive autophagy. When macrophages overexpressed miR‐124‐3p, the induction of the M2 phenotype was suppressed. Luciferase reporter assay proved the combination of circRps5 and miR‐124‐3p.

**Conclusion:**

This study identifies that circRps5 carried by ADSC‐Exos promotes macrophage M2 polarization through miR‐124‐3p. These findings provide valuable insights into the mechanism of ADSC‐Exos for treating refractory diabetic wounds, laying a solid theoretical groundwork for future clinical development.

## Graphical abstract

The morphology of adipose‐derived stem cells‐derived exosomes was analyzed by transmission electron microscopy and identified using nanoparticle tracking analysis and exosome markers.

## 1 INTRODUCTION

According to estimates, impaired wound healing affects approximately 25% of diabetes patients, often leading to lower limb amputations.^1^ The high glucose environment promotes the formation of biofilms, making the treatment of diabetic wounds challenging.^2^ Macrophages have been extensively studied in the context of the immune system of diabetic patients and chronic wound models. One reason is their ability to produce and release cytokines under the influence of the surrounding microbiota, thereby coordinating the transition from the inflammatory phase to the proliferative phase.^3^, ^4^ In acute wounds, M1 macrophages are replaced by M2 macrophages as the inflammatory phase subsides.^5^ However, in the case of diabetic wounds, M1 macrophages continue to dominate the wound microenvironment, which is considered a key factor contributing to the delayed healing of diabetic wounds.^6^ This has been demonstrated in previous research articles. For instance, stimulation of M2 macrophage polarization and fibroblast activation with IL‐25 has been shown to promote the healing of diabetic wounds.^7^ Studies using diabetic rat models and human monocytic THP‐1 cells have discovered that insulin promotes the transition of M1 to M2 macrophages during the wound healing process, thereby improving the healing of diabetic wounds.^8^ The novel hydrogel biomaterial for the treatment of diabetic foot ulcers effectively accelerates wound healing in type 1 diabetic rat and db/db mouse models by promoting the recruitment of M2 macrophage populations.^9^ This transition shifts the state of wound ulcers from the inflammatory phase to the proliferative and remodeling phases.

As the number of diabetes patients continues to increase,^10^ there is a growing demand for the management of chronic wounds, necessitating further research to uncover methods for accelerating the healing of diabetic wounds and improving patients' quality of life. Adipose‐derived stem cells (ADSCs) are adult mesenchymal stem cells that exist in adipose tissue, possessing self‐renewal capacity, multilineage differentiation potential, and genetic stability.^11^ Due to their abundant source and straightforward isolation methods, they have demonstrated significant potential for application in skin regeneration and tissue engineering in recent years.^12^, ^13^ Previous studies have found that enhancing ADSC autophagy promotes the repair of diabetic wounds.^14^ ADSCs therapy promotes vascularization in diabetic foot ulcer rats, thereby accelerating the healing of skin wounds.^15^ Isolation of extracellular vesicles derived from ADSCs has been shown to facilitate angiogenesis in a mouse model of diabetic wounds, thereby improving wound healing in diabetic mice.^16^ After induction of low oxygen conditions, the survival and proliferation capacity of ADSCs is significantly enhanced compared to normoxic conditions, further shortening the time required for wound healing and preventing scar formation in a mouse model of skin defects.^17^, ^18^ These studies suggest significant advantages of ADSCs in the treatment of diabetic wounds, although the underlying mechanisms of repair have not been fully elucidated.

Previous studies have discovered that exosomes from ADSCs transport proteins or nucleic acid substances to target cells, mediating intercellular signaling and regulating damaged tissues.^19^, ^20^ Compared with ADSCs, exosomes have the advantages of stable biological function, convenient transportation and management, and low antigenicity. A study found that hypoxic preconditioned ADSCs can promote the transformation of macrophage M1−M2 in brain injury by releasing exosomes carrying high levels of circRps5.^21^ miR‐124‐3p is a downstream target of circRps5 and downregulation of miR‐124‐3p can promote macrophage M2 polarization in nerve injury.^22^ It is proposed that ADSCs exosomes regulate macrophage polarization to promote diabetic wound healing via the circRps5/miR‐124‐3p axis.

In this study, a diabetic mouse wound model was constructed, and exosomes from ADSCs were isolated to elucidate the potential mechanisms by which ADSCs promote wound healing. This will provide theoretical support for the use of ADSCs or their exosomes in the treatment of nonhealing diabetic wounds.

## 2 MATERIAL AND METHODS

### 2.1 Exosome isolation

ADSCs were incubated in the primary mesenchymal stem cell medium (iCell) containing additives (iCell), 10% fetal bovine serum (FBS, BioMed) and 1% penicillin‐streptomycin solution, and inoculated into T75 square flasks. ADSCs cells were cultured under hypoxic conditions (1% O_2_, 5% CO_2,_ and 94% N_2_) for 24 h, and the ADSCs supernatant was collected for exosomes extraction. The supernatant was centrifuged at 4°C and 3000*g* for 10 min, and the exosome extraction reagent (Keygentec) was added to the new supernatant and mixed well. The mixture was allowed to stand at 4°C for 2 h, centrifuged at 12,000*g* for 20 min, the supernatant was discarded, and the exosome pellet was resuspended in PBS.

### 2.2 Exosome identification

Exosomes were fixed in 2.5% glutaraldehyde at 4°C, dehydrated with gradient alcohol, and embedded in epoxy resin. Sections were stained with uranyl acetate and citrate acid lead, and captured under a transmission electron microscope (TEM, JEOL). Exosomes were dissolved in RIPA buffer (Biosharp) and quantified using a bicinchoninic acid assay (BCA) protein concentration assay kit (Biosharp). The exosome markers (CD9, CD63, CD81, and HSP70) were detected by immunoblotting as described below. The particle size and concentration were measured using ZetaView nanoparticle tracking analysis (NTA, Particle Metrix).

### 2.3 Preparation of different exosomes

To investigate the role of circRps5 in exosome regulation, exosomes from differently treated ADSCs were used in subsequent studies. ADSCs were transfected with circRps5 overexpression (OE‐circRps5) plasmids to increase the level of circRps5, while empty plasmids were regarded as negative controls (OE‐NC). In addition, GW4869 (20 μM, MCE) was added to another group of ADSCs to inhibit the secretion of exosomes. Briefly, the mixture of plasmids and Lipofectamine 2000 transfection reagent (Invitrogen) diluted in serum‐free medium was added to the ADSCs, and after incubation at 37°C and 5% CO_2_ for 6 h, complete medium with or without GW4869 was added. Subsequently, the ADSCs cultured for 24 h were continued to be cultured under hypoxic conditions for 24 h, and the supernatant was collected to extract exosomes as aforementioned.

### 2.4 Diabetic wound model

A total of 36 C57BL/6 mice (8 weeks, male, Yangzhou University Comparative Medicine Center) were reared for 1 week in an SPF animal laboratory at 21−23°C and humidity of 60%−65%. Six mice were given a normal diet, and the rest were fed a high‐fat diet for 1 week, and then intraperitoneally injected with streptozotocin (STZ, 50 mg/kg, Bomeibio) to induce diabetes mellitus (DM). The tail vein blood glucose was detected by a blood glucose meter (Yuwell), and the value > 16.7 mmol/L indicated that the DM standard was met, and the high‐fat diet was continued for 4 weeks. Thereafter, mice were anesthetized with 3% isoflurane, the hair on the back of the shoulder was shaved, and an incision with a diameter of 1 × 1 cm was made in the middle. DM mice (*n* = 30) were randomly divided into five groups: model, model + Exos, model + GW4869‐Exos, model + OE‐circRps5‐Exos, model + OE‐NC‐Exos. In the treatment group, different exosomes (2 mg/100 μL PBS) were injected into four central points on the edge of the wound. Mice in the control group were injected with an equal volume of PBS solution. After 28 days of treatment, the mice in each group were euthanized by an overdose of anesthesia.

### 2.5 H&E staining

The tissue at the wound was fixed with 4% paraformaldehyde solution (Sigma), dehydrated with gradient alcohol, and embedded in paraffin. The samples were cut into 4‐µm sections, rehydrated with gradient alcohol, and stained with hematoxylin solution (Zhanyun) for 5 min, followed by eosin (Zhanyun) for 2 min at room temperature. Sections were gradually dehydrated with gradient alcohol and permeabilized with xylene. Specimens were photographed under a microscope (Leica).

### 2.6 Masson staining

Sections were rehydrated with gradient alcohol, stained with Weigert iron hematoxylin for 5 min, and differentiated with acidic ethanol for 10 s. Then sections were treated with the Masson blue solution (Servicebio) for 1 min followed by ponceau red magenta dyeing solution for 5 min. Subsequently, sections were washed with the phosphomolybdic acid solution for 10 min and counterstained with the aniline blue dyeing solution for 5 min, the results were observed after dehydration under a light microscope. Collagen volume was quantified using ImageJ software.

### 2.7 ELISA

The levels of wound injury‐related factors Collagen (COL)‐Ⅰ, COL‐Ⅲ, α‐smooth muscle actin (α‐SMA) and C‐reactive protein (CRP), and inflammation‐related factors interleukin (IL)‐6, tumor necrosis factor (TNF)‐α, IL‐4 and IL‐10 in mouse serum were determined by commercial kits (Mlbio). Whole blood was left at room temperature for 1 h and centrifuged at 1000*g* for 20 min at 4°C, and the supernatant was taken as the samples. The serial dilution of the standard, the co‐incubation with the biotinylated antibody, the labeling of the horseradish peroxidase, the addition of the chromogenic reagent, and the stop solution were all carried out according to the operating instructions. The optical density of each well was measured with a microplate reader (Molecular Devices) at a wavelength of 450 nm.

### 2.8 Macrophage culture and handling

Raw264.7 cells were seeded in a 6‐well plate and incubated in DMEM (Biosharp) containing 10% FBS (Thermo Fisher) in a 5%CO_2_ atmosphere at 37°C. Serum‐free medium was used to synchronize the cells for 24 h, and the cells were induced into the M1 type using lipopolysaccharide (LPS, 500 nM, Sigma) along with 25 mmol/L glucose for 24 h. Similar to the grouping of mice, M1 polarized raw264.7 cells (5 × 10^6^) were co‐incubated with 100 μg/mL different exosomes (Exos, GW4869‐Exos, OE‐circRps5‐Exos, OE‐NC‐Exos) for 48 h.

### 2.9 Quantitative real‐time PCR (RT‐qPCR)

TriZol (Biosharp) was used to extract RNA from exosomes, tissues, or cells. Isopropanol was added to the supernatant obtained after the pre‐cooled chloroform treatment, and the RNA pellet was obtained by centrifugation at 10,000 rpm for 15 min at 4°C. The first‐strand cDNA was synthesized with a reverse transcription kit (Crondabio). Primers were designed and synthesized by Shanghai Sangon company. The PCR reaction was performed with a LightCycler 480 II system with a SYBR Green Real‐Time PCR kit (Crondabio). Data were normalized to 18 s RNA or β‐actin using the ^△△^ *C*_t_ method. Primer sequences are listed in Table 1.

**Table 1:** Primer sequences for quantitative real‐time PCR.

Gene name	Primer forward (5′ to 3′)	Primer Reverse (5′ to 3′)
circ‐Rps5	CCGAGTGCCTTGCAGATG	AAAGCAGCCTCACGAGCC
18 s RNA	AGGCGCGCAAATTACCCAATCC	GCCCTCCAATTGTTCCTCGTTAAG
miR‐124‐3p	CAAGTAAGGCACGCGG	CTCAACTGGTGTCGTGGA
U6	CTCGCTTCGGCAGCACA	AACGCTTCACGAATTTGCGT
CD86	TGTTTCCGTGGAGACGCAAG	TTGAGCCTTTGTAAATGGGCA
iNOS	GTTCTCAGCCCAACAATACAAGA	GTGGACGGGTCGATGTCAC
CD206	CTCTGTTCAGCTATTGGACGC	CGGAATTTCTGGGATTCAGCTTC
Arg‐1	CTCCAAGCCAAAGTCCTTAGAG	AGGAGCTGTCATTAGGGACATC
β‐actin	GTCCCTCACCCTCCCAAAAG	GCTGCCTCAACACCTCAACCC

Abbreviation: iNOS, inducible nitric oxide sythase.

### 2.10 Immunoblotting

Proteins were harvested from the tissue homogenate or cell lysate using RIPA, quantified using a BCA kit, and denatured at 95°C. Electrophoresis was performed to separate the proteins and hybridized membranes (Millipore) were obtained through a transfer system. The membranes were blocked in skimmed milk and incubated with primary antibodies (Proteintech). HRP‐conjugated secondary antibody (Proteintech) was used to visualize the antigen. Following development with the ECL reagent (Millipore), the gray values of blots were analyzed with ImageJ software.

### 2.11 Immunofluorescence

Deparaffinized sections were treated with 0.1% Triton x‐100 (BioFROXX), blocked with 5%BSA for 1 h, and incubated with CD86 (Abcam) + F4/80 (Proteintech) or CD206 (Proteintech) + F4/80 primary antibody (all diluted at 1: 500) overnight at 4°C. Then they were incubated with Cy3‐conjugated goat anti‐mouse (Servivebio) or FITC‐conjugated goat anti‐rabbit secondary antibody (Servivebio) 37°C for 1 h in the dark. For raw264.7 cells, they were fixed in 4% paraformaldehyde for 30 min, blocked with 5% BSA, and incubated with LC3B primary antibody (Proteintech) and Cy3‐labeled goat anti‐rabbit antibody (Servivebio). Sections were counterstained with Hoechst33258 (Beyotime) for 10 min and treated with antifade mounting medium (Beyotime). Immunostaining was observed under a fluorescence microscope.

### 2.12 Macrophage phagocytosis

PKH67 (Umibio) marks exosomes to observe their phagocytosis by macrophages. 50 μL of freshly prepared PKH26 linker working solution diluted 10 times with Diluent C was incubated with 100 μg of exosomes in the dark for 10 min. Afterward, the mixture was washed with PBS, mixed with the exosome isolation reagent for 2 h, and then centrifuged at 12,000*g* at 4°C for 20 min. The supernatant was discarded, and the exosome pellet was cocultured with raw264.7 cells. After 48 h, the supernatant was removed, the cells were washed twice with PBS, and intracellular and extracellular exosomes were observed under a microscope.

### 2.13 Flow cytometry

After co‐incubating with exosomes for 48 h, raw264.7 cells were harvested and resuspended in PBS. The cell density was adjusted to 10^6^, and 100 μL of Staining Buffer was added. The cells were incubated with CD86 and CD206 antibodies (BioLegend) at 4°C in the dark for 30 min. The cells were washed twice with PBS, and the cells were resuspended in Staining Buffer, and detected by FACS Aria^TM^ III flow cytometry immediately.

### 2.14 Luciferase reporter assay

HEK‐293T cells were plated into 48‐well plates at a density of 1.5 × 10^4^ cells/well. The mixture of the pmiGLO luciferase reporter vector and miRNA mimics was cotransfected into HEK‐293T cells using Lipofectamine 2000 (Invitrogen). Cells were harvested after transfection for 24 h,^23^ and then the luciferase activity assay was performed by the dual‐luciferase reporter assay system (Promega).

### 2.15 Statistical analysis

Data are presented as means ± standard deviations (SD) of three experiments. One‐way or two‐way analysis of variance with Tukey's post hoc test was used to compare multiple groups. Statistical analysis was performed using SPSS 26.0 software and the value of *p* < .05 was considered significant.

## 3 RESULTS

### 3.1 Identification of ADSC exosomes (ADSC‐Exos)

**Figure 1:**
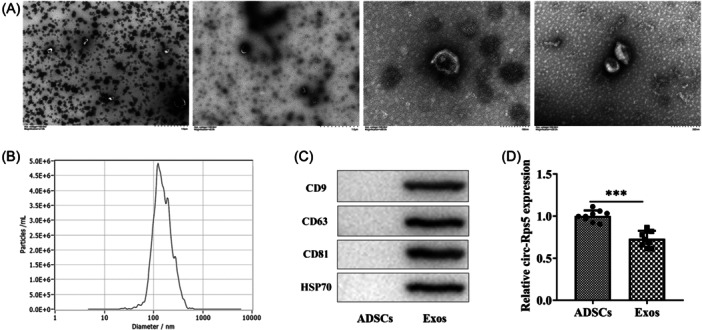
Identification of ADSC exosomes. (A) The morphology of ADSC‐derived exosomes was analyzed by transmission electron microscopy. (B) NTA measures the particle size distribution of ADSC‐derived exosomes. (C) Immunoblotting analysis of known exosome markers CD9, CD63, CD81, and HSP70. (D) The expression level of circRps5 in ADSC exosomes was detected by RT‐qPCR. ***p < .001. ADSC, adipose‐derived stem cells; NTA, nanoparticle tracking analysis; RT‐qPCR, quantitative real‐time PCR.

Exosomes from ADSCs presented a cup‐ or sphere‐shaped morphology according to TEM (Figure 1A). NTA analysis identified the mean diameter of exosome was 125.2 nm (Figure 1B). Exosome markers CD9, CD63, CD81, and HSP70 were positive in isolated exosomes and negative in ADSCs (Figure 1C). circRps5 expression was identified in exosomes at slightly lower levels than in ADSCs (Figure 1D).

### 3.2 ADSC‐Exos promote wound healing in diabetic mice

**Figure 2:**
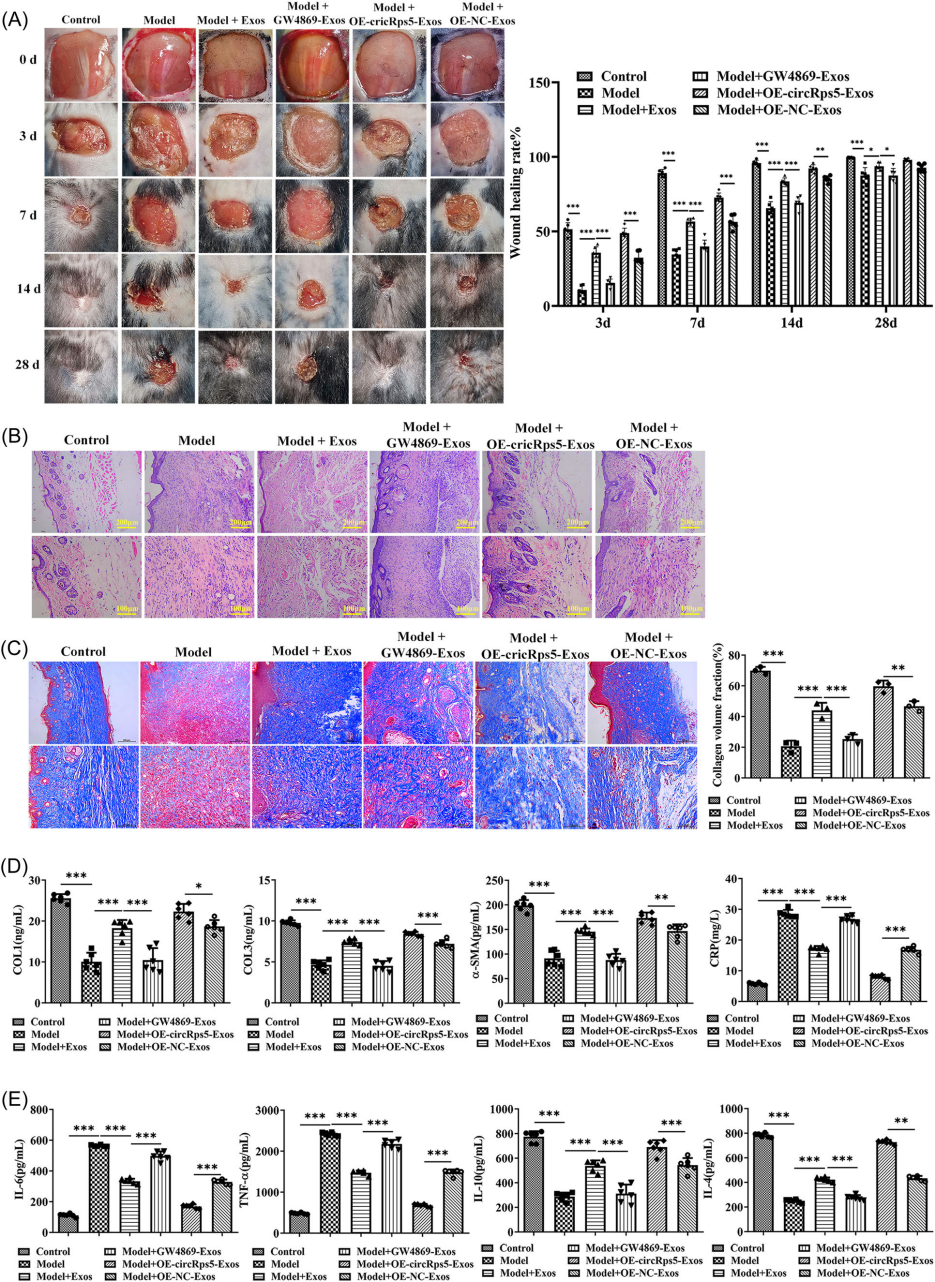
ADSC exosomes promote wound healing in diabetic mice. (A) Photos of the wound healing of mice in each group from 0 to 28 days, and the quantitative results of wound healing rate. (B) H&E staining results of mouse wounds. (C) Masson staining results of mouse wounds and quantitative results of collagen. (D) The levels of wound injury‐related factors COL‐Ⅰ, COL‐Ⅲ, α‐SMA, and CRP and (E) inflammation‐related factors IL‐6, TNF‐α, IL‐10, and IL‐4 in mouse serum were determined using ELISA. *p < .05, **p < .01, ***p < .001. α‐SMA, α‐smooth muscle actin; ADSC, adipose‐derived stem cells; CRP, C‐reactive protein; IL, interleukin; TNF, tumor necrosis factor.

The wound healing in the model group was slower than that in the control group within 28 days. Exosomes from ADSCs promoted wound healing, which could be reversed by GW4869. Compared with the model + OE‐NC group, OE‐cricRps5 effectively promoted wound healing (Figure 2A). Compared with the control group, there was a large number of lymphocytes and neutrophils in the wound tissue of the model group, and the number of new blood vessels and fibroblasts decreased. Fibroblasts increased and inflammatory cells decreased in the model + exos group, while GW4869 reversed the effect. The status of the model + oe‐circRps5 group was closer to that of the control group (Figure 2B). The tissue collagen fiber density of the model group was significantly lower compared to the control group, and the collagen hyperplasia was obvious. The degree of fibrosis in the model + exos group was significantly higher than that in the model group. GW4869 reversed the effect of exos, whereas the collagen production in the model + OE‐circRps5 group was superior to that in the model + exos group (Figure 2C). The levels of wound injury and inflammation‐related factors in serum were detected by ELISA. In the model group, the levels of COL‐Ⅰ, COL‐Ⅲ, and α‐SMA were significantly downregulated, and the level of CRP was upregulated. The model + exos group upregulated the levels of COL‐Ⅰ, COL‐Ⅲ, and α‐SMA, and downregulated the level of CRP, which was more obvious in the model + OE‐circ‐Rps5 group, and the treatment of GW4869 reversed this effect (Figure 2D−E).

### 3.3 ADSC‐Exos affect macrophage polarization

**Figure 3:**
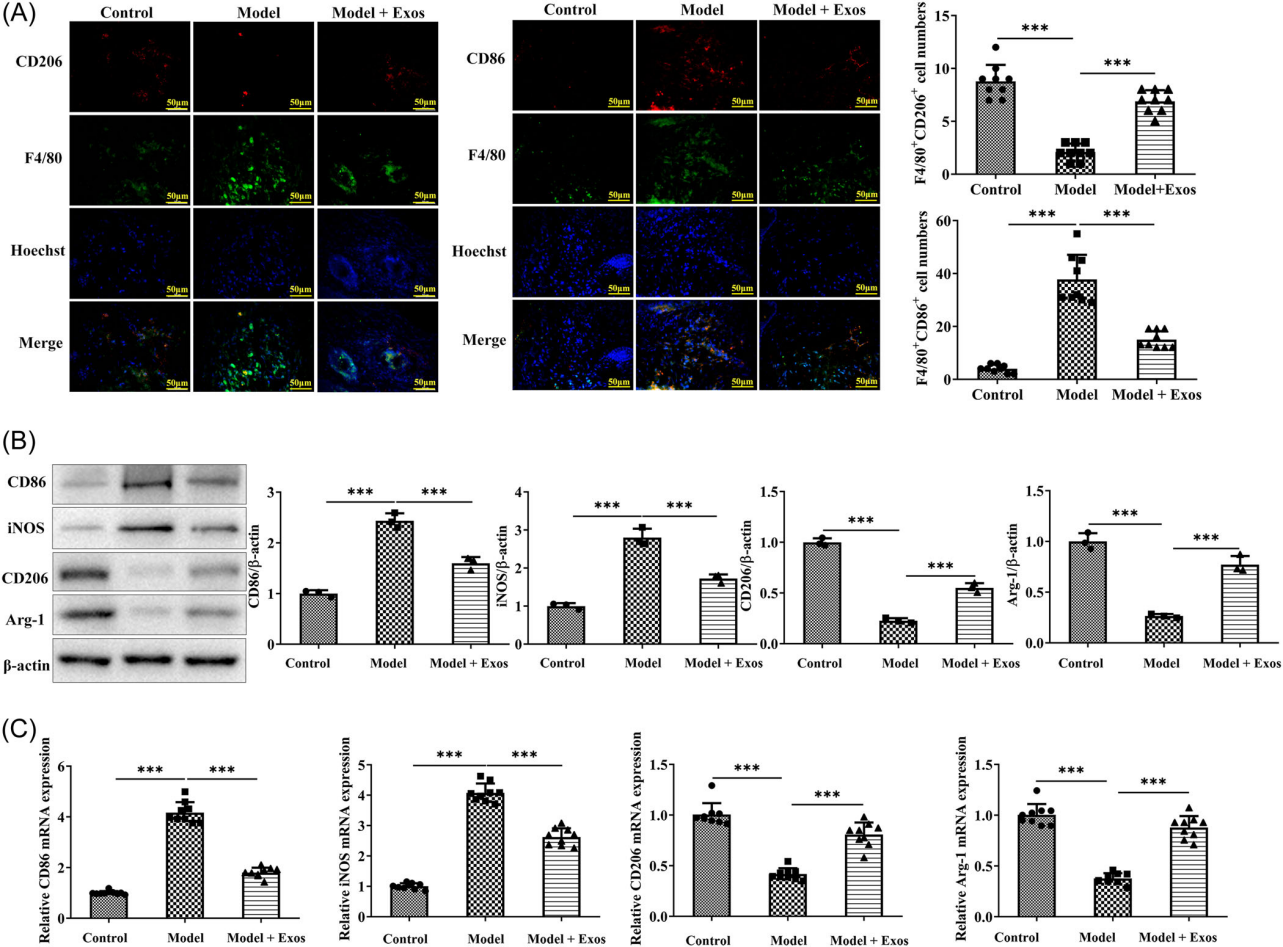
ADSC exosomes affect macrophage polarization. (A) Immunofluorescence indicates the levels of M1 marker CD86 and M2 marker CD206. (B) Immunoblotting and (C) RT‐qPCR demonstrate the protein and mRNA levels of CD86 and iNOS, along with CD206 and Arg‐1. ***p < .001. ADSC, adipose‐derived stem cells; iNOS, inducible nitric oxide sythase; RT‐qPCR, quantitative real‐time PCR.

Immunofluorescence results showed that compared with the control group, the expression of CD86 in the model group was increased, and that of CD206 was decreased. Exosome treatment could reduce the expression of CD86 and increase that of CD206 (Figure 3A). The results of immunoblotting (Figure 3B) and RT‐qPCR (Figure 3C) demonstrated that compared with the control group, the protein and mRNA levels of CD86 and inducible nitric oxide sythase (iNOS) in the model group increased, while CD206 and Arg‐1 decreased. Compared with the model group, exosome treatment reduced the protein and mRNA levels of CD86 and iNOS, and increased those of CD206 and Arg‐1.

### 3.4 circRps5 carried by ADSCs exosomes regulates macrophage polarization

**Figure 4:**
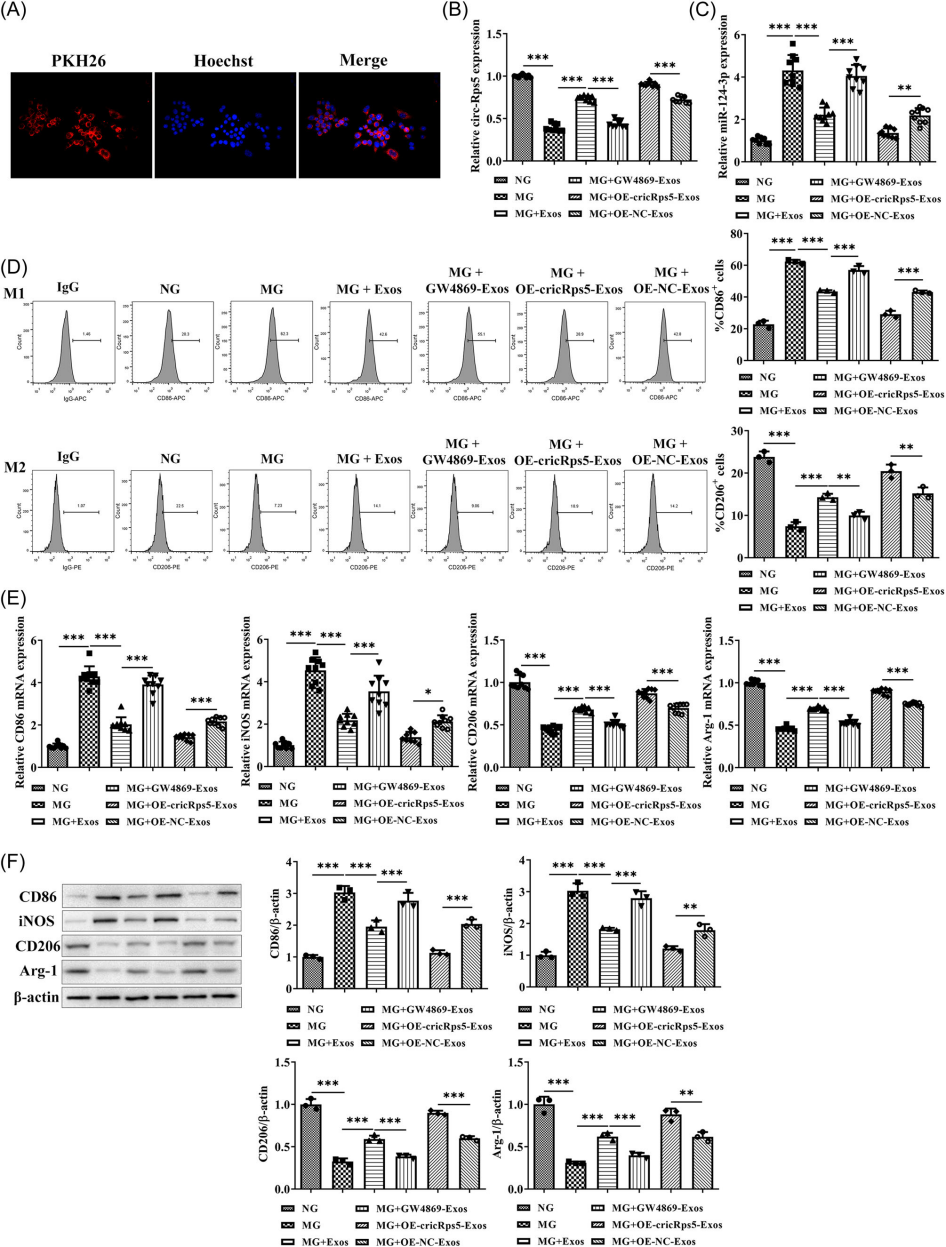
circRps5 carried by ADSCs exosomes regulates macrophage polarization. Raw264.7 cells were co‐treated with high glucose and exosomes from different ADSCs. (A) The internalization of PKH‐26‐labeled ADSC‐Exos into raw264.7 cells. (B) The mRNA level of circRps5 and (C) miR‐124‐3p was detected by RT‐qPCR. (D) The proportion distribution of M1 and M2 macrophages was detected by flow cytometry. (E) RT‐qPCR and (F) Immunoblotting demonstrate the mRNA and protein levels of CD86 and iNOS, along with CD206 and Arg‐1. *p < .05, **p < .01, ***p < .001. ADSC, adipose‐derived stem cells; iNOS, inducible nitric oxide sythase; mRNA, messenger RNA; RT‐qPCR, quantitative real‐time PCR.

PKH26‐labeled exosomes were distributed in the cytoplasm, indicating that exosomes were phagocytized by raw264.7 cells (Figure 4A). MG induced a significant downregulation of circRps5 level in raw264.7 cells, whereas miR‐124‐3p level was increased. Normal exosomes increased the level of circRps5 and reduced miR‐124‐3p, which was more obvious in the OE‐circRps5‐exos group. GW4869 treatment reversed the effect of normal exosomes (Figure 4B,C). Furthermore, MG promoted M1 polarization in raw264.7 cells, which showed an increase in the proportion of CD86‐positive cells and a decrease in the proportion of CD206‐positive cells. Normal exosomes promoted M2 polarization, which was more pronounced in the OE‐circRps5‐exos group, while GW4869 reversed the effect of normal exosomes (Figure 4D). In addition to flow cytometry, protein and mRNA levels of M1 and M2 markers in raw264.7 cells were detected using RT‐qPCR (Figure 4E) and immunoblotting (Figure 4F). The protein and mRNA levels of CD86 and iNOS in the MG group increased, while CD206 and Arg‐1 decreased. Normal exosome treatment reduced the protein and mRNA levels of CD86 and iNOS, and increased those of CD206 and Arg‐1, which was more obvious in the OE‐circRps5‐exos group, while GW4869 reversed the effect of normal exosomes.

### 3.5 circRps5 carried by ADSCs exosomes relieves excessive autophagy

**Figure 5:**
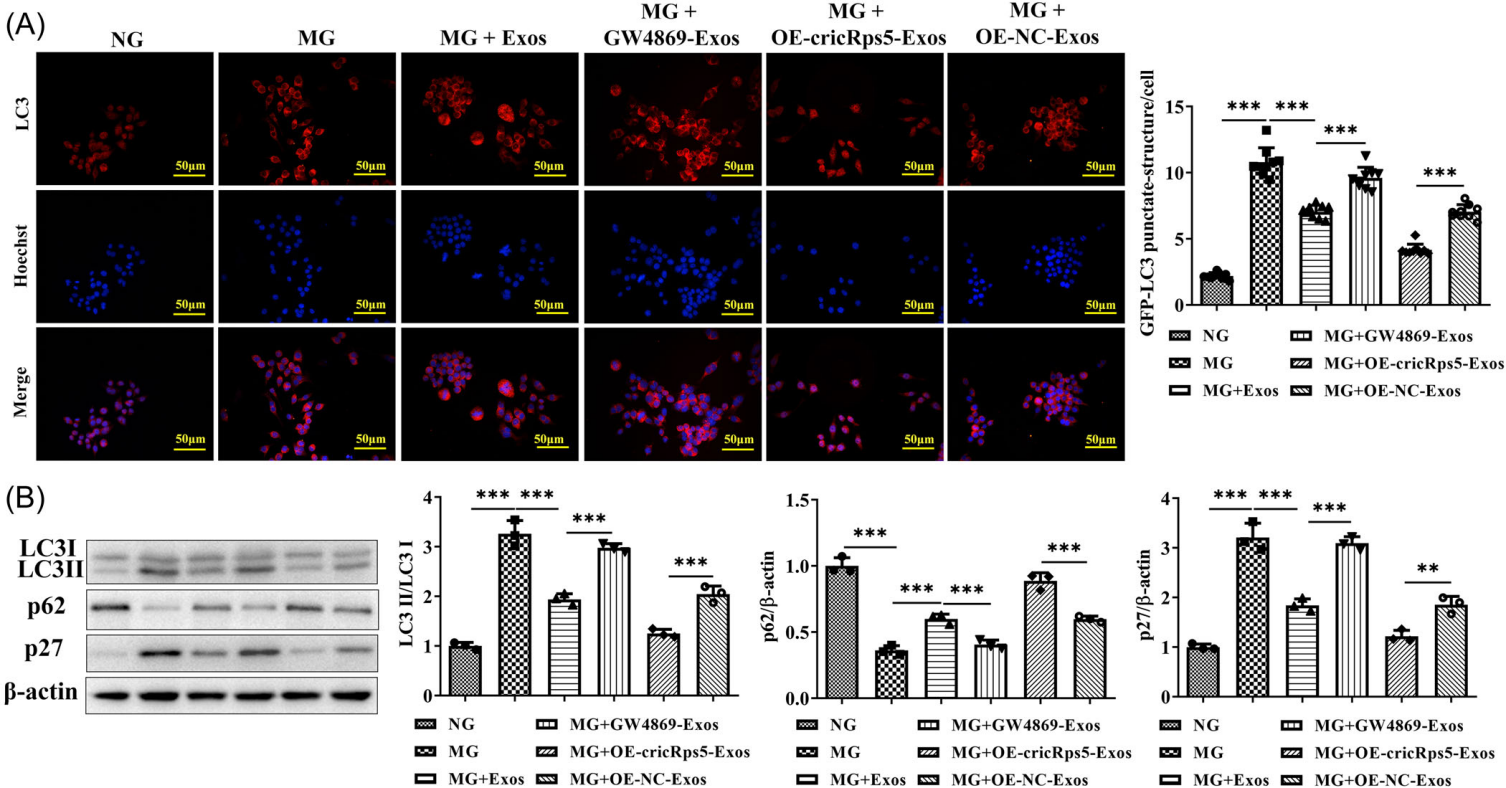
circRps5 carried by ADSCs exosomes relieves excessive autophagy. (A) Immunofluorescence indicates the levels of LC3. (B) Immunoblotting demonstrates the protein levels of autophagy‐related proteins. **p < .01, ***p < .001. ADSC, adipose‐derived stem cells.

MG induced a significant elevation of LC3 level in raw264.7 cells, and normal exosomes reduced the level of LC3 compared to the MG group. GW4869 treatment reversed the effect of normal exosomes and the MG + OE‐circRps5‐exos group showed a more obvious reduction of LC3 (Figure 5A). Compared with the NG group, p27, and LC3II/LC3I proteins were increased and p62 proteins were decreased in the MG group. Exosomes caused the protein contents of p27 and LC3II/LC3I to decrease, and the protein content of p62 to increase. Compared with the MG + OE‐NC‐exos group, OE‐circRps5‐exos significantly reduced the protein content of p27 and LC3II/LC3I and significantly increased the protein content of p62. The addition of GW4869 reversed the effect of normal exosomes (Figure 5B).

### 3.6 miR‐124‐3p is downstream of circRps5 to regulate macrophage polarization

**Figure 6:**
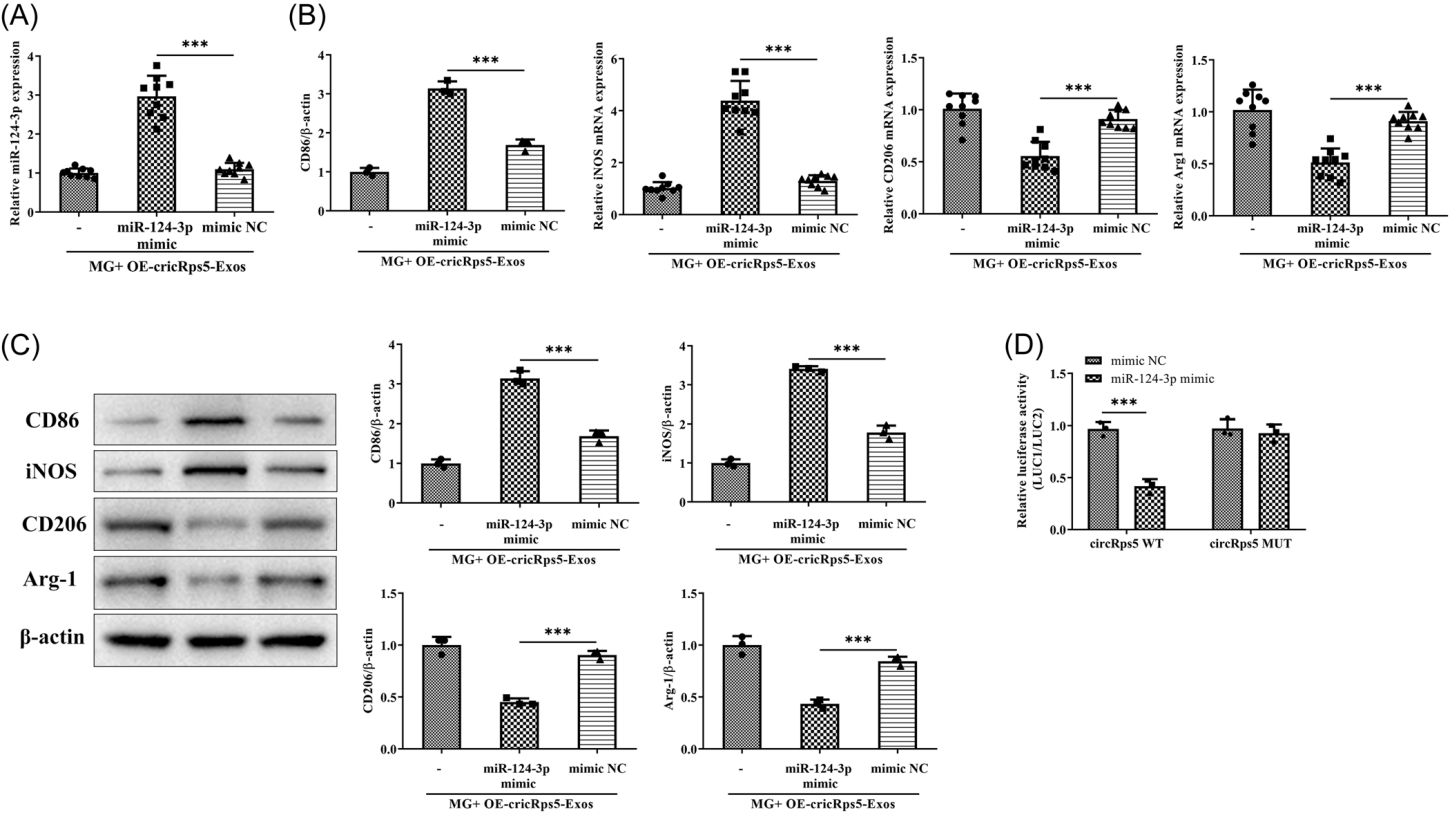
miR‐124‐3p is downstream of circRps5 to regulate macrophage polarization. (A) Raw264.7 cells were transfected to increase miR‐124‐3p levels. (B) RT‐qPCR and (C) immunoblotting analysis of the mRNA levels of CD86, iNOS, CD206, and Arg‐1 in the transfected cells. (D) Luciferase reporter assay demonstrates that circRps5 could bind to miR‐124‐3p. ***p < .001. iNOS, inducible nitric oxide sythase; RT‐qPCR, quantitative real‐time PCR.

To identify the mediating of miR‐124‐3p in regulation, raw264.7 cells were induced miR‐124‐3p overexpression by transfection. After treatment with MG + OE‐circRps5‐exos, miR‐124‐3p was still highly upregulated, as shown by RT‐qPCR results (Figure 6A). Treatment of miR‐124‐3p overexpression promoted M1 polarization, which was reflected in the increase of CD86 and iNOS, and the decrease of CD206 and Arg‐1 (Figure 6B,C). Luciferase reporter assay demonstrated that miR‐124‐3p mimic significantly reduced the luciferase activity of wild‐type circRps5, but did not affect the mutant circRps5. This indicated that circRps5 could bind to miR‐124‐3p (Figure 6D).

## 4 DISCUSSION

Diabetes is an escalating health concern characterized by high blood sugar resulting from cellular insulin resistance and deficiency.^24^ Diabetic foot trauma is a serious chronic complication of diabetes, where soft tissue wounds and ulcers arise from a combination of neuropathy, peripheral vascular disease, and hyperglycemia.^25^ There is evidence that patients with diabetic foot trauma exhibit higher levels of inflammatory factors.^26^ The mainstay of treatment is to address extrinsic factors such as repeated trauma, ischemia, and infection, and to optimize glycemic control.^27^ While conventional treatments like debridement and wound dressings have proven inadequate, novel therapies have emerged, including bioengineered skin substitutes, hyperbaric oxygen therapy, electrical stimulation pulse therapy, and stem cell therapy, among others.^27^, ^28^

Stem cell‐derived exosomes have gained significant traction in recent years, presenting a promising alternative to existing treatments.^29^ Numerous original studies have reported the impact of various stem cell exosomes on diabetic wounds.^30^, ^31^ These exosomes, carrying diverse proteins, metabolites, DNA, and RNA, selectively induce specific signals to regulate the immune response and function of recipient cells.^32^ Among stem cells, ADSC‐Exos stand out due to their abundant source and less invasive extraction process compared to bone marrow mesenchymal stem cells (BMSCs).^33^

This study focuses on circRps5, a noncoding RNA carried by ADSC‐Exos, which plays a pivotal role in regulating diabetic wound healing. The circular structure of circRps5 enhances its stability compared to linear RNA,^34^ and ADSC‐Exos carry a broader array of ncRNAs and signaling pathways than BMSC‐Exos, making them more effective in promoting wound healing through signaling regulation.^18^

Furthermore, the study identifies that circRps5 carried by ADSC‐Exos promotes macrophage M2 polarization through miR‐124‐3p. A clinical study reveals that various microRNAs are increased in patients with diabetic complications.^35^ In diabetic wounds, an abundance of circulating blood monocytes differentiates into M1 macrophages,^36^ which secrete proinflammatory factors TNF‐α and IL‐6, contributing to inflammation and necrotic cell debris removal. Hyperglycemia hinders the inflammatory functions of macrophages, particularly their phagocytic activity. In contrast, M2 macrophages secrete factors, such as IL‐4 and IL‐10, that facilitate collagen synthesis and wound epithelialization.^6^, ^37^ ADSC‐Exos rectify the imbalanced macrophage phenotypes, thereby reducing persistent inflammation in diabetic wounds.

## 5 CONCLUSION

In summary, this study provides valuable insights into the mechanism of ADSC‐Exos for treating refractory diabetic wounds, laying a solid theoretical groundwork for future clinical development. Whether utilized independently or in combination with biomaterials, exosome therapy holds significant clinical value and promising application prospects.

## AUTHOR CONTRIBUTIONS

Dongjing Yin and Guoliang Shen contributed to the concept, experiments, and analysis. Dongjing Yin contributed to the draft and Guoliang Shen provided critical opinions. They approve the final version of the manuscript.

## CONFLICT OF INTEREST STATEMENT

The authors declare no conflict of interest.

## ETHICS STATEMENT

Operations were strictly performed according to the National Institute of Health Guide for the Care and Use of Laboratory Animals. This study was supported by The First Affiliated Hospital of Soochow University (No. IACUC‐20220630‐06).

## Data Availability

The data sets used and/or analyzed during the current study are available from the corresponding author upon reasonable request.
